# Tracking the Subtle Mutations Driving Host Sensing by the Plant Pathogen *Streptomyces scabies*

**DOI:** 10.1128/mSphere.00367-16

**Published:** 2017-03-01

**Authors:** Samuel Jourdan, Isolde M. Francis, Benoit Deflandre, Rosemary Loria, Sébastien Rigali

**Affiliations:** a InBioS—Centre for Protein Engineering, University of Liège, Institut de Chimie, Liège, Belgium; b Department of Biology, California State University, Bakersfield, California, USA; c Department of Plant Pathology, University of Florida, Gainesville, Florida, USA; Loyola University Chicago

**Keywords:** host sensing, *Streptomyces*, host-pathogen interactions, phytopathogens, plant pathogens, scab disease

## Abstract

The acquisition of genetic material conferring the arsenal necessary for host virulence is a prerequisite on the path to becoming a plant pathogen. More subtle mutations are also required for the perception of cues signifying the presence of the target host and optimal conditions for colonization. The decision to activate the pathogenic lifestyle is not “taken lightly” and involves efficient systems monitoring environmental conditions. But how can a pathogen trigger the expression of virulence genes in a timely manner if the main signal inducing its pathogenic behavior originates from cellulose, the most abundant polysaccharide on earth? This situation is encountered by *Streptomyces scabies*, which is responsible for common scab disease on tuber and root crops. We propose here a series of hypotheses of how *S. scabies* could optimally distinguish whether cello-oligosaccharides originate from decomposing lignocellulose (nutrient sources, saprophyte) or, instead, emanate from living and expanding plant tissue (virulence signals, pathogen) and accordingly adapt its physiological response.

*Streptomyces* species are well-known soil-dwelling bacteria that actively participate in the recycling of organic matter mainly originating from plant residual biomass through diverse enzymatic systems (cellulases, amylases, xylanases, etc.) (1). Only a few members of this genus have evolved from a saprophytic to a pathogenic lifestyle, with *Streptomyces scabies* as a model organism. Plant-pathogenic *Streptomyces* bacteria are responsible for scab disease, which can have severe consequences for production yields of economically important root and tuber vegetables (2). Thaxtomin A, the main virulence determinant produced by *S. scabies*, inhibits the cellulose biosynthesis process in plant tissues expanding underground (3, 4). Cellobiose and cellotriose have been reported as the main carbohydrates triggering thaxtomin production and therefore the onset of the pathogenic behavior of *S. scabies* (5, 6). Proteins of the cello-oligosaccharide-mediated induction of thaxtomin production consist of the CebEFG-MsiK ATP-binding cassette (ABC) transporter system (7) (Fig. 1). Control of the thaxtomin biosynthesis genes at the transcriptional level involves a locking-unlocking system with the cellulose utilization repressor CebR as the locking key and the activator TxtR as the opening key (8, 9). Despite the fact that the key players in the thaxtomin induction pathway have been recently characterized in detail, it is still unclear how *S. scabies* manages to sense cello-oligosaccharides as a virulence signal and not as a nutrient source. Here we propose a series of features that could possibly permit *S. scabies* to avoid behaving as a pathogen if the conditions are more appropriate for a saprophytic lifestyle.

**FIG 1:**
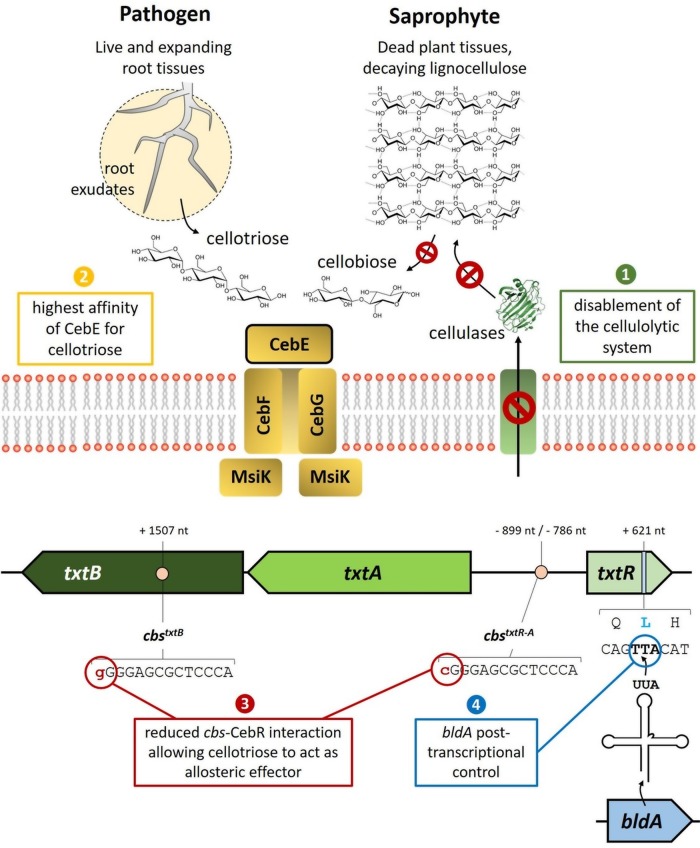
Genetic and physiological features predicted for the adaptation of *S. scabies* to a pathogenic lifestyle built upon the perception of cello-oligosaccharides. Factors: 1, disabling of the cellulolytic system; 2, increased affinity of the transporter sugar-binding CebE for cellotriose (root exudates) instead of cellobiose (breakdown of cellulose); 3, fine-tuned control of *txtR* expression by CebR; 4, posttranscriptional control of* txtR* by *bldA*, encoding the leucyl-tRNA for the rare UUA codon. nt, nucleotides.

## Disabling of the cellulolytic system.

Organisms able to actively participate in the generation of virulence elicitors are perceived as evolutionarily more sophisticated than opportunistic pathogens that only passively wait for the looked-for signal to occur. However, when the induction of the pathogenicity system is mainly built upon the sensing of molecules originating from the most abundant polymer in organic soils (cellulose), the first step on the path to virulence should certainly differ from participation in the genesis of the signal. Having based its phytotoxin production on the use of cello-oligosaccharides is also very intriguing, as the signal is, first of all, an important nutrient source that mostly originates from the hydrolysis of perishing plant material. Therefore, it does not seem appropriate to use these cello-oligosaccharides as a trigger for virulence if the releasing hosts are dead. A priority for *S. scabies* should entail avoiding the utilization of cellulose of decomposing plants if this species was to evolve to essentially behave as a pathogen. Indeed, *S. scabies* is unable to grow and use any type of cellulose when it is provided as the main carbon source, which prevents this organism from enzymatically generating its own virulence elicitors (5; unpublished data). How *S. scabies* disabled its complete extracellular enzymatic system dedicated to cellulose utilization is currently unknown, and providing an answer to this question will most likely highlight a key mechanism in “how a saprophytic organism can become a plant pathogen.” The mutations involved must affect key proteins in induction and/or secretion mechanisms, as *S. scabies* has a full set of genes for celluloclastic and cellulolytic systems (*cel* genes; Table 1), including enzymes belonging to the glycosyl hydrolase 9 (GH9), GH12, GH48, and GH74 families, which, in streptomycetes, are found only in species with high cellulolytic activity (10). This paradox is reinforced by the abundance of perfect CebR-binding sites (CBSs) found in *S. scabies* (14 CBSs upstream of genes encoding putative cellulases; Table 1), as the same study indeed showed a positive correlation between the number of CBSs found in different *Streptomyces* species and their ability to hydrolyze cellulose (10). Interestingly, when cellulose-containing media are supplied with alternative carbon sources that do not impose carbon catabolite repression, a cellulase activity can be detected, confirming that the cellulolytic system of *S. scabies* is functional (11; S. Jourdan, unpublished data). Thus, *S. scabies* would need to first use other carbon sources to initiate spore germination and early growth before considering cellulose as a possible nutritive substrate.

**TABLE 1:** Predicted cellulose and cello-oligosaccharide utilization genes in *S. scabies*^a^

Category and gene	Function	CebR box^b^	Position(s)
Cellulose, cello-oligosaccharide extracellular degradation			
* scab1101*	Secreted GH (probable β-glucosidase), GH5 family	ND	
* scab5981*, *celB*	CelB, secreted cellulase B (endoglucanase), GH12 family with CBM2 domain	TGGGAGCGCTCCCA	−82
* scab8871*, *cel1*	Secreted endoglucanase, GH9 family with CBM4 domain	TGGGAGCGCTCCCA	−90
* scab16431*	Putative cellulase CelA1 (endoglucanase), GH6 family	TGGGAGCGCTCCCA	−113
		TGGaAGCGCTtCCA	−128
		TGGGAGCGCTtCCg	−264
* scab17001*	Secreted cellulase (β-1,4-exocellulase), GH6 family with CBM2 domain	TGGGAGCGCTCCCA	−130
		TGGaAcCGCTCCCA	−283
* scab17011*	Putative secreted cellulase, GH48 family with CBM2 domain	TGGGAGCGCTCCCA	−311
		TGGaAcCGCTCCCA	−158
* scab17021*	Putative secreted cellulase, GH74 family with CBM2 domain	ND^c^	
* scab21081*, *celS2*	CelS2 secreted lytic polysaccharide monooxygenase with CBM2 domain (possible *bldA* regulation)	TGGGAGCGCTCCCA	−177
* scab51081*	Secreted cellulase (beta-glucosidase?), GH5 family with CBM2 domain (possible *bldA* regulation)	TGGGAGCGCTCCCA	−54
* scab78881*	Secreted GH, GH5 family with CBM2 and fn3 domains	TGGGAGCGCTCCCA	−85
* scab86271*	Putative secreted hydrolase, GH5 family	ND	
* scab86311*	Putative secreted endoglucanase, GH5 family	ND	
* scab86471*	Putative endoglucanase (cellulase), GH5 family with CBM2	ND	
* scab89741*	Secreted cellulose-binding protein with chitin (CBM3) binding domain	TGGGAGCGCTCCCA	−116
* scab90061*	Putative cellulase, expansion, peptidoglycan-binding domain (CBM63)	TGGGAGCGCTCCCA	−201
* scab90081*	Secreted cellulase B, GH12 family with CBM2 domain	TGGGAGCGCTCCCA	−60
* scab90091*	Cellulose 1,4-β-cellobiosidase, GH48 family with CBM2 domain	TGGGAGCGCTCCCA	−105
		TGGGAGCGgTtCCc	−248
		cGGGAGCGCTCCCA	−674
* scab90101*	Cellulose 1,4-β-cellobiosidase, GH6 family with CBM2 domain	TGGGAGCGCTCCCA	−761
		TGGGAGCGgTCCCa	−618
		cGGGAGCGCTCCCA	−192
Transport, intracellular degradation, and regulation			
* scab57721*, *bglC*	BglC, putative intracellular β-glucosidase, GH1 family	TGGaAGCGCTCCCA	−13
* scab57751*, *cebE*	CebE, cellobiose/cellotriose binding component of CebEFG-MsiK ABC-type transporter	TGGGAGCGCTCCCA	−130
* scab57761*, *cebR*	CebR, cellulose/cello-oligosaccharides utilization repressor, thaxtomin biosynthesis expression repressor	TGGGAGCGCTCCCA	−496
Phytotoxin biosynthesis			
* scab31781*, *txtB*	TxtB, thaxtomin synthetase B	gGGGAGCGCTCCCA	+1507
* scab31791*, *txtA*	TxtA, thaxtomin synthetase A	cGGGAGCGCTCCCA	−899
* scab31801*, *txtR*	TxtR, cellobiose-dependent thaxtomin biosynthesis transcriptional activator	cGGGAGCGCTCCCA	−786

a Abbreviations: CBM, cellulose-binding domain; fn, fibronectin; GH, glycosyl hydrolase; ND, not detected.

b Lowercase letters indicate nucleotides that do not match with the CebR consensus sequence TGGGAGCGCTCCCA.

c Probably regulated by the two CebR boxes identified upstream of the adjacent *scab17011* gene.

## How to distinguish identical molecules from live or dead plants.

The presence of cello-oligosaccharides in an environment devoid of potential living hosts could result from the degradation of lignocellulose derived from dead plant tissue by the action of cellulolytic microorganisms rather than by *S. scabies* itself, as discussed above. Although *S. scabies* contains cellulases that are transcriptionally and/or enzymatically silent, this species possesses an efficient CebEFG-MsiK ABC-type transport system (7) with a very high affinity (in the nanomolar range) of CebE for cellobiose and cellotriose—respectively, 100 to 1,000 times higher than the affinity constants measured for CebE of the highly cellulolytic species *Streptomyces reticuli* (12, 13). This suggests that *S. scabies* would be adapted to behave as a commensal in soils with decaying plant cell walls. Nevertheless, *S. scabies* must have developed a mechanism that allows it to distinguish whether a source of cellobiose/cellotriose is derived from living or dead plant tissue in order to avoid counterproductive production of the phytotoxin thaxtomin. Although cellobiose, because of its lower cost than cellotriose, is commonly used as an inducer of thaxtomin production under laboratory conditions, the trisaccharide has been reported to be a much better elicitor than the disaccharide (5). Interestingly, Johnson et al. showed that cellotriose (but not cellobiose) is present in the root exudates of growing radish seedlings, as well as in tobacco cell suspensions (5). Instead, cellobiose is the main by-product released by the action of the cellulolytic system, while only marginal cellotriose is produced by the enzymatic degradation of cellulose (14). These observations suggest that cellotriose could be the signal molecule that indicates the presence of a growing root network, whereas cellobiose would signal dead plant cell walls on which to feed. Expanding plant tissues releasing cellotriose constitute not only the site of invasion of the pathogen but also the site of action of thaxtomin. Johnson et al. also showed that the addition of thaxtomin resulted in an increase in the amount of cellotriose released by growing tissues (5). Thus, small amounts of cellotriose would be sufficient to initiate the production of thaxtomin, which, in turn, would lead to the release of a greater amount of cellotriose, which would trigger massive production of the phytotoxin. Thaxtomin itself, instead of the cellulolytic system, would be responsible for generating the inducer of its own biosynthesis, as previously proposed by Johnson et al. This hypothesis implies that *S. scabies* would have evolved a mechanism able to monitor very low concentrations of cellotriose emanating from root exudates. This seems to be the case, as we have shown that CebE of *S. scabies* has higher binding affinity for the trisaccharide (dissociation constant [*K*_*d*_] for cellotriose, ~2 nM) than for the disaccharide (*K*_*d*_ for cellobiose, ~14 nM), which is unusual for *Streptomyces* ABC-type sugar-binding components (12, 15, 16). Whether the presence of cellotriose and the absence of cellobiose are constants in exudates from roots and tubers commonly damaged by *S. scabies* remains to be demonstrated. We are also currently investigating if the greater affinity of CebE for cellotriose than for cellobiose is a specific feature of pathogenic *Streptomyces*. Site-directed mutagenesis will allow the identification of residues involved in this unusually high substrate affinity and bias toward the trisaccharide.

## Imperfect CebR *cis*-acting elements associated with virulence genes.

In *Streptomyces* and other *Actinobacteria* species, the regulator CebR (also named CelR) represses the expression of *cel* genes and the genes for cello-oligosaccharide uptake (7, 10, 17–19), which in *S. scabies* are also required for induction of thaxtomin production (8), by binding to CBSs with the 14-bp TGGGAGCGCTCCCA motif as the consensus palindromic sequence (Table 1). Genes that belong to the same regulon do not display the same expression level under both repressed and activated conditions. Indeed, for the proper response of a metabolic pathway, the expression of a certain category of genes has to be tightly inhibited while the basal expression of others is required. In the case of the CebR regulon in *S. scabies*, one can imagine that genes involved in the interpretation of cello-oligosaccharides as a virulence signal might be differently regulated than those strictly considering cellulose by-products as nutrient sources. This differential expression pattern is, in great part, imposed by the distribution of one or more CebR *cis*-acting elements with different affinities in the gene’s upstream region (Table 1). Of the 18 genes known or presumed to encode extracellular cellulases in *S. scabies*, 14 possess at least one perfect palindromic CBS in their upstream region (Table 1). Efficient activation of their transcription thus requires cellobiose, the best allosteric effector of CebR, while cellotriose only partially prevents its DNA-binding ability (8). The import of small amounts of cellotriose by the CebEFG-MsiK transporter would therefore have a much weaker effect on the derepression of *cel* genes than the thaxtomin biosynthetic (*txtA* and *txtB*) and regulatory (*txtR*) genes, which are more weakly bound by CebR because of the presence of imperfect *cis*-acting elements (Fig. 1) (8). We therefore anticipate that cellotriose uptake from root exudates will first activate the expression of *txtR*, *txtA*, and *txtB*, while cellobiose import from lignocellulose degradation will trigger the expression of genes of the cellulolytic system.

## *bld**A*-dependent developmental posttranscriptional control.

The pathway-specific transcription factor TxtR is absolutely required for the activation of thaxtomin biosynthetic genes (7). This essential gene for the induction of *S. scabies* pathogenicity is not only controlled by CebR at the transcriptional level but also possesses a TTA codon at nucleotide position 621 (20) (Fig. 1); TTA codons are extremely rare in GC-rich *Streptomyces* species. The UUA codon in *Streptomyces* mRNA can only be translated into a leucine by the leucyl-tRNA encoded by the *bldA* gene. Posttranscriptional regulation by *bldA* is a common feature associated with physiological and morphological differentiation processes in all *Streptomyces* bacteria (21–23). The inactivation of *bldA* results in a so-called bald (nonsporulating) phenotype with concomitant impaired production of the specialized metabolites (antibiotics, siderophores, etc.). Induction of *bldA* transcription occurs in order to allow the translation of mRNA encoding proteins that enable adaptation to transient stress conditions and/or occur during late exponential growth, that is, when *Streptomyces* bacteria switch from primary to secondary metabolism and trigger their developmental processes (21). This additional posttranscriptional control mediated by *bldA* would prevent *S. scabies* from producing thaxtomin even in the presence of cello-oligosaccharides unless other factors (stress, population density, life cycle stage, etc.) affecting *bldA* expression are present, indicating the proper timing to switch from the saprophytic to the pathogenic lifestyle.

## Concluding remarks and perspectives.

Previous reviews have listed and discussed in detail the acquired large genetic material—at the gene or gene cluster level—for *S. scabies* to behave as a plant pathogen (24, 25). Although these major virulence determinants are now easier to identify as we have entered the era of high-speed and cost-efficient genome sequencing, finding the discrete but equally essential genetic changes necessary for the transition from a saprophytic to a pathogenic lifestyle remains a challenging task. Our search for the subtle genetic causes that would possibly explain the physiological adaptation of *S. scabies* includes mutations that (i) modify the affinity of the CebE sensor for the most appropriate virulence cue, cellotriose; (ii) weaken the interaction strength of the transcriptional repressor CebR for its *cis*-acting elements; and (iii) impose posttranscriptional control by the use of the rare TTA codon, ensuring that toxin production correlates with the developmental transition. The mutations associated with disabling of the cellulolytic system remain enigmatic at the current state of our knowledge but might include some of those cited above. For instance, *scab51081* and *scab21081* (*celS2*) are possible targets of BldA posttranscriptional regulation, as they have a TTA codon at nucleotide positions 13 and 22, respectively. In addition, tight CebR-mediated repression of the expression of *scab16431*, *scab17001*, *scab17011*, *scab90091*, and *scab90101* is also suggested, as evidenced by multiple CebR boxes in their upstream region (Table 1). The hypotheses presented and discussed here require further experimental validation and are probably only a part of all the minor changes that appeared in the ancestors of pathogenic *Streptomyces* species once they acquired the thaxtomin biosynthesis cluster.
